# Herpes zoster as a vaccine-preventable risk factor increases the risk of dementia: A nested case-control study in Chinese population

**DOI:** 10.1080/21645515.2026.2681253

**Published:** 2026-06-29

**Authors:** Binshan Jiang, Yi Yan, Chengzhong Xu, Xuanzi Zhao, Fei Qi, Songtao Xu, Weizhong Yang, Xiaojun Liu, Luzhao Feng

**Affiliations:** a School of Population Medicine and Public Health, Chinese Academy of Medical Sciences and Peking Union Medical College, Beijing, China; b State Key Laboratory of Respiratory Health and Multimorbidity, Key Laboratory of Pathogen Infection Prevention and Control (Peking Union Medical College), Ministry of Education, Beijing, China; c Yichang Centre for Disease Control and Prevention, Yichang, Hubei, China; d Department of Dermatology, Peking University People’s Hospital, Beijing, China; e National Health Commission Key Laboratory of Medical Virology and Viral Diseases, National Institute for Viral Disease Control and Prevention, Chinese Center for Disease Control and Prevention, Beijing, China

**Keywords:** Herpes zoster, varicella zoster virus, exposure, dementia, Alzheimer’s disease, vascular dementia, nested case-control study

## Abstract

Herpes zoster, a vaccine-preventable disease caused by the reactivation of varicella-zoster virus, has been linked to an increased risk of dementia in high-income countries. However, evidence from populations with low vaccination coverage remains limited. To address this gap, we conducted a nested case-control study using electronic health records from Yichang, China. A total of 51,843 incident dementia patients aged 50 y and older were matched with 338,877 controls by sex, age, and visit date. A documented clinical history of herpes zoster was significantly associated with an elevated risk of all-cause dementia (adjusted odds ratio [aOR] = 1.48; 95% confidence interval [CI]: 1.41–1.56), with the highest risk observed among individuals with herpes zoster involving the central nervous system (aOR = 1.59, 95%CI: 1.46–1.73). Among the dementia cases with prior HZ diagnosis, both Alzheimer’s disease (aOR = 1.44, 95%CI: 1.14–1.79) and vascular dementia (aOR = 1.82, 95%CI: 1.49–2.21) showed increased risks following herpes zoster infection, with vascular dementia demonstrating a stronger association and more rapid progression (median time from first herpes zoster diagnosis to dementia onset: 1.5 y versus 2.2 y for Alzheimer’s disease). The findings, derived from a population with minimal herpes zoster vaccination coverage, identify herpes zoster as a potentially modifiable risk factor for dementia. These also underscore the potential dual public health benefit of herpes zoster vaccination in preventing both acute infection and long-term cognitive decline.

## Introduction

The varicella-zoster virus (VZV), a neurotropic alphaherpesvirus, establishes lifelong latency in the dorsal root ganglia following primary infection (chickenpox).^1^ Over 95% of adults aged 50 y and older are seropositive for VZV, reflecting a widespread risk of viral reactivation globally.^2^ This risk manifests clinically as herpes zoster (HZ) or shingles, and increases substantially with advancing age due to the decline in cell-mediated immunity, a phenomenon known as immunosenescence.^3^ Emerging epidemiological evidence suggests that HZ episodes are associated not only with acute dermatological manifestations but with a range of short- and long-term risks of systemic diseases. These include postherpetic neuralgia, myocardial infarction, cerebrovascular events, and notably, neurodegenerative conditions such as dementia and Parkinson’s disease.^4–8^

Dementia, a neurodegenerative disorder characterized by the damage and loss of neurons and their synaptic connections in the brain, constitutes another major age-related health burden. Its prevalence increases progressively with advancing age, making it a leading cause of years of life lost and disability-adjusted life years globally. In China, an estimated 16.99 million individuals live with Alzheimer’s disease (AD) and related cognitive impairments, accounting for nearly 30% of the global dementia population. The disease burden continues to rise rapidly, with the crude incidence rate increasing by 242.5% between 1990 and 2021.^9^

The intricate interplay between HZ and dementia remains a vibrant area of ongoing epidemiological research. Studies have attempted to elucidate this association by examining VZV infection, HZ occurrence, the effects of antiviral therapy, and correlations with dementia biomarkers or diagnoses.^10^ However, whether dementia is linked to previous HZ is a subject of much debate, even in the meta-analysis, and most existing data originate from high-income countries.^6,11,12^ Although a definitive conclusion remains elusive, accumulating evidence increasingly leans toward affirming this association. Recent meta-analyses suggest that HZ may represent a modifiable risk factor for dementia in older adults; this hypothesis is strengthened by observations of reduced dementia incidence among populations vaccinated against HZ.^13^ With the increasing availability and utilization of safe and effective HZ vaccines, these findings may influence public health strategies, particularly relevant for China’s approach to integrated prevention of multiple age-related conditions.

HZ has been a vaccine-preventable disease in China since 2020; however, the coverage remains notably low among the target population. The increasing burden of HZ and its debilitating complications poses a growing challenge to the healthcare systems, underscoring an urgent public health concern.^14^ This study primarily aims to address critical evidence gaps by investigating the association between history of HZ and subsequent dementia diagnoses among Chinese adults aged 50 y and older. A secondary objective is to examine this association differs by specific HZ categories – including HZ ophthalmicus (HZO), complicated HZ, and HZ with central nervous system (CNS) involvement – and dementia subtypes, specifically Alzheimer’s disease and vascular dementia. We conducted this investigation using a large, population-based, real-world dataset representing individuals living with dementia in low- and middle-income countries. This Chinese cohort offers a unique and previously underrepresented perspective, providing essential evidence to inform future research and public health value in similar settings.

## Patients and methods

### Data source and study setting

This nested case-control study extracted data from the Yichang Healthcare Big Data Center in Hubei Province, central China, which maintains a nationally recognized advanced electronic medical record (EMR) system covering over 3.9 million permanent residents of Yichang City.^15^ This medium-sized city has an urbanization rate of 66% and represents a typical prefecture-level setting in China. Its economic development and healthcare infrastructure aligns closely with the national average for such cities, falling intermediate between major metropolitan centers and rural counties. Approximately 45% of the population aged 50 y or older, slightly exceeding the national average, rendering Yichang a particularly pertinent sentinel site for studying age-related disease burdens including herpes zoster and dementia.

Using unique encoded identifiers, the platform comprehensively integrates demographic characteristics, clinical diagnostic records coded according to the International Classification of Diseases, Tenth Revision (ICD-10), prescription data, laboratory results, and health expenditure information, establishing lifelong electronic health profiles for all residents. The study period spanned from January 1, 2016, to May 31, 2025, excluding the preliminary test phase in 2015 to ensure data stability. The validity of HZ and dementia diagnostic coding within this database has been established through its use in prior epidemiological studies.^16–18^ The study protocol was reviewed and approved by the Institutional Review Board of the Chinese Academy of Medical Sciences and Peking Union Medical College, in accordance with the Declaration of Helsinki. The requirement for informed consent was waived due to the use of completely deidentified data.

### Study population and participant selection

Data from January 1, 2016 of the population with outpatient and inpatient diagnoses were screened. Individuals lacking documented sex, date of birth, or diagnosis date, or with any diagnosis date preceding their birth date, were disqualified. Dementia cases were defined as individuals aged 50 y or older with a first-time incident outpatient or inpatient diagnosis of dementia. Dementia was identified using specific ICD-10 codes (Table 1 in the Supplement). To enhance diagnostic accuracy and minimize misclassification inherent in electronic health record data, we employed a composite case definition. Specifically, dementia cases were included if they had either (1) at least two recorded diagnoses of dementia on separate dates, or (2) at least one diagnosis accompanied by a prescription for dementia-specific medication (e.g., cholinesterase inhibitors or memantine). Based on the initial dementia diagnosis code, cases were further classified into AD, vascular dementia (VD), or other dementia subtypes. Cases with dementia attributed to trauma or with a history of congenital dementia were excluded. The index date was defined as the date of the first dementia or trauma diagnosis.

**Table 1.** Selected baseline characteristics of patients diagnosed with dementia and matched trauma controls.

Characteristic	Overall (N = 390720)	Dementia Patients(N = 51843)	Control Group (N = 338877)	*p* value
	n	n	n	
Sex				.005
Male	198289 (50.75%)	26608 (51.32%)	171681 (50.66%)	
Female	192431 (49.25%)	25235 (48.68%)	167196 (49.34%)	
Age at index date (years) mean (std)	69.0 (8.91)	72.3 (9.37)	68.5 (8.73)	
Age group				<.001
50–64	118041 (30.21%)	10403 (20.07%)	107638 (31.76%)	
65–79	221522 (56.70%)	29489 (56.88%)	192033 (56.67%)	
80–102	51157 (13.09%)	11951 (23.05%)	39206 (11.57%)	
Ethnicity (N = 52835)				<.001
Han	46678 (88.35%)	32681 (87.71%)	13997 (89.86%)	
Minority	6157 (11.65%)	4578 (12.29%)	1579 (10.14%)	
Education background (N = 46099)				.034
Junior high school and below	34722 (75.32%)	24473 (75.63%)	10249 (74.59%)	
High (Vocational) school education	8456 (18.34%)	5818 (17.98%)	2638 (19.20%)	
Bachelor’s degree or above	2921 (6.34%)	2068 (6.39%)	853 (6.21%)	
Marital status (N = 50132)				<.001
Married	42222 (84.22%)	29520 (83.17%)	12702 (86.78%)	
Discoverture (single widowed or divorced)	7910 (15.78%)	5975 (16.83%)	1935 (13.22%)	
Disabled				<.001
Intelligence disability	27 (0.01%)	25 (0.05%)	2 (0%)	
Other disabilities	1652 (0.42%)	1199 (2.31%)	429 (0.13%)	
None	389068 (99.58%)	50620 (97.64%)	338448 (99.87%)	
Exposure				<.001
Simple HZ	5405 (1.38%)	1049 (2.02%)	4356 (1.29%)	
HZ with CNS-involvement	3258 (0.83%)	674 (1.30%)	2584 (0.76%)	
Complicated HZ	1177 (0.30%)	191 (0.37%)	986 (0.29%)	
HZO	261 (0.07%)	42 (0.08%)	219 (0.06%)	
None	380619 (97.4%)	49887 (96.23%)	330732 (97.6%)	
Age at herpes zoster (years) mean (std)	69.0 (8.63)	71.6 (9.25)	68.3 (8.35)	<.001
Antiviral drug				<.001
Yes	4828 (1.24%)	1017 (1.96%)	3811 (1.12%)	
No	385892 (98.8%)	50826 (98.04%)	335066 (98.9%)	
Length of follow up (years) mean (std)	2.16 (1.92)	2.30 (2.01)	2.13 (1.89)	.001
Underlying medical condition				
Smoking history	3231 (0.83%)	2637 (5.09%)	594 (0.18%)	<.001
Alcohol consumption	3235 (0.83%)	2041 (3.94%)	1194 (0.35%)	<.001
Obesity	173 (0.04%)	24 (0.05%)	149 (0.04%)	.903
Hypertension	104565 (26.8%)	14314 (27.6%)	90251 (26.6%)	<.001
Diabetes mellitus	37854 (9.69%)	4677 (9.02%)	33177 (9.79%)	<.001
Dyslipidemia	28822 (7.38%)	3044 (5.87%)	25778 (7.61%)	<.001
Depression	1737 (0.44%)	329 (0.63%)	1408 (0.42%)	<.001
Blind	7908 (2.02%)	943 (1.82%)	6965 (2.06%)	<.001
Deaf	4463 (1.14%)	505 (0.97%)	3958 (1.17%)	<.001
Cardiovascular disease	22256 (5.70%)	3397 (6.55%)	18859 (5.57%)	<.001
Cerebrovascular accident	65293 (16.70%)	9654 (18.6%)	55639 (16.42%)	<.001
Peripheral vascular disease	4430 (1.13%)	705 (1.36%)	3725 (1.10%)	<.001
COPD	70039 (17.93%)	5838 (11.3%)	64201 (18.9%)	<.001
Chronic kidney disease	8296 (2.12%)	1130 (2.18%)	7166 (2.11%)	.347
Liver disease	19874 (5.09%)	2422 (4.67%)	17452 (5.15%)	<.001
Tumor	19655 (5.03%)	1866 (3.60%)	17789 (5.25%)	<.001
Herpes simplex virus	2229 (0.57%)	153 (0.30%)	2076 (0.61%)	<.001
Parkinson	1854 (0.47%)	505 (0.97%)	1349 (0.40%)	<.001
Number of risk factor*				<.001
0	254475 (65.13%)	32638 (62.96%)	221837 (65.46%)	
1–2	125823 (32.20%)	17143 (33.07%)	108680 (32.07%)	
>2	10422 (2.67%)	2062 (3.98%)	8360 (2.47%)	

HZ: herpes zoster, CNS: central nervous system, HZO: herpes zoster ophthalmicus, COPD: chronic obstructive pulmonary disease.

*Number of risk factor including the diagnosis of hearing impairment, depression, hypertension, alcohol abuse, obesity, diabetes, dyslipidemia, visual impairment, and ischemic heart disease before the index date.

For each case, up to eight controls were selected using propensity score matching from individuals with an incident diagnosis of trauma and no recorded diagnosis of dementia prior to or on the index date. Controls were matched to cases based on age (within a 2-year caliper), sex, and index date (within a 30-day caliper). The earliest index date was set to January 1, 2017, to ensure at least one year of medical record history for all participants from the study start date (January 1, 2016) until their outcome event. Individuals with a history of HZ vaccination were excluded from both the case and control groups.

### Assessment of exposure and covariates

The primary exposure of interest was a documented history of HZ episode, identified by the ICD-10 code B02, occurring before the index date. To ensure a more specific exposure definition, HZ infections were further categorized into ocular, CNS, complicated, and simple skin infections based on relevant diagnostic codes (Supplementary Table 1). The follow-up duration was defined as the length of year from the earliest recorded date of any HZ diagnoses to the index date.

Demographic covariates included age at the index date and sex. Covariates of underlying health condition and risk factors were selected based on established dementia risk factors from the 2024 report of the Lancet Standing Commission.^19^ These included hearing impairment, depression, hypertension, alcohol abuse, obesity, diabetes, dyslipidemia, visual impairment, and ischemic heart disease, all identified via ICD-10 codes recorded before the index date (Supplementary Table 1).

### Statistical analysis

All statistical analyses were performed using R Statistical Software (version 4.3.2). Baseline characteristics were summarized as means and standard deviations (SDs) for continuous variables and proportions for categorical variables. Group comparisons were conducted using Student’s t-tests, Chi-squared tests, or Fisher’s exact test, as appropriate. Logistic regression models were used to estimate the propensity score, the association between HZ exposure and dementia risk with results expressed as odds ratios (ORs) and 95% confidence intervals (CIs). Both univariable and multivariable models were constructed, with the latter adjusted for age group, sex, and the number of pre-existing dementia risk factors (categorized as 0, 1–2, or >2). Subgroup analyses were conducted by sex and dementia subtypes. Separate regression models were established for all-cause dementia, AD, and VD.

To evaluate robustness, two sensitivity analyses were performed. First, individuals were excluded if they had intellectual disability, or a history of HSV infection or Parkinson’s disease prior to the index date. Given that both HSV and VZV are herpesviruses and their cutaneous manifestations require differential diagnosis. HSV has been independently linked to driving dementia.^20^ Parkinson’s disease, another common neurological disorder in the elderly that can progress cognitive impairment leading to dementia, has also been linked to VZV exposure according to meta-analyses.^11^ Additionally, to account for potential impacts of the COVID-19 pandemic on patterns of co-infection and healthcare-seeking behavior, subgroup analyses were conducted based on the index date before (2016–2019) and after January 1, 2020 (2020–2025). Multivariable logistic regression models were fitted separately within each period to assess the consistency of the association between HZ and dementia across the contexts.

To assess the time-dependent impact of HZ on dementia progression, a survival analysis was conducted among the dementia cases with prior HZ diagnosis. Kaplan-Meier curves were plotted to visualize cumulative dementia incidence stratified by HZ categories, and differences were compared using the log-rank test. A univariable Cox proportional hazards model was fitted to estimate hazard ratios for dementia associated with different HZ categories.

All statistical tests were two-sided, and a *p*-value < .05 was considered statistically significant.

## Results

### Baseline characteristics of the study population

A total of 390,720 individuals were included in this analysis, comprising 51,843 dementia patients and 338,877 matched controls, with a median of 6 matched trauma controls per case. The quality of matching was verified using standardized mean differences and standardized pairwise distances, with absolute values below 0.1 indicating negligible differences between groups^21^ (supplementary Table 2). The baseline characteristics of the study participants are summarized in Table 1 and supplementary Table 3. The mean age of participants was 69.0 y (SD 8.91), and patients in the dementia cohort were older. Among the dementia patients, 2,891 were initially identified with AD and 3,595 with VD. Both the AD and VD groups had a higher mean age of 76 y compared to those with other dementia types. The percentage of male sex was slightly higher in the dementia group (51.3% vs. 48.7%). The prevalence of most medical condition was higher in the dementia group than in the control group.

### Association of herpes zoster and dementia

In the univariable analysis, a history of HZ was significantly associated with an increased risk of dementia (OR = 1.47, 95% CI: 1.40–1.55). When stratified by infection type, HZ with CNS-involvement showed the strongest association (OR = 1.59, 95% CI: 1.46–1.73), followed by simple HZ (OR = 1.47, 95% CI: 1.37–1.58) and complicated HZ (OR = 1.21, 95% CI: 1.04–1.42). HZO was not significantly associated with dementia (OR = 1.21, 95% CI: 0.87–1.69). After adjusting for age groups, sex, and the number of concurrent dementia risk factors, the association between HZ exposure and all-cause dementia (aOR = 1.48, 95% CI: 1.41–1.56) remained significant and was slightly increased. The associations for different HZ anatomical sites were generally consistent with the univariable results, shown in Table 2. In addition, male sex was associated with a higher risk (aOR = 1.11, 95% CI: 1.09–1.13) compared to female sex. Increasing age was a strong risk factor; compared to the under-65 group, the aOR was 1.59 (95% CI: 1.55–1.63) for the 65–79 age group and 3.17 (95% CI: 3.08–3.26) for the 80-and-over group. In age-stratified analyses, the association was consistent across all age groups, with aORs for individuals aged 50–64 y, 65–79 y, and aged 80 y and older were 1.77 (95% CI: 1.56–2.00), 1.38 (95% CI: 1.06–1.80) and 1.54 (95% CI: 1.15–2.06) respectively (Supplementary Table 4). Having more than two dementia risk factors was also associated with a significantly increased risk (aOR = 1.52, 95% CI: 1.44–1.60) compared to having no risk factors.

**Table 2.** Risk of all-cause dementia, Alzheimer’s disease and vascular dementia for herpes zoster with four categories by conditional logistic analysis.

Variable	All Dementia (N = 390720)		Alzheimer’s Disease (N = 19280)		Vascular Dementia (N = 24621)	
	OR (95% CI)	*P* value	OR (95% CI)	*P* value	OR (95% CI)	*P* value
All-type Herpes Zoster	1.47 (1.40–1.55)	<.001	1.32 (1.06–1.65)	.015	1.72 (1.41–2.09)	<.001
Simple Herpes Zoster	1.47 (1.37–1.58)	<.001	1.37 (1.02–1.84)	.034	1.80 (1.37–2.35)	<.001
Herpes Zoster with CNS-involvement	1.59 (1.46–1.73)	<.001	1.33 (0.89–1.97)	.160	1.67 (1.20–2.32)	.002
Complicated Herpes Zoster	1.21 (1.04–1.42)	.016	0.81 (0.36–1.82)	.606	1.94 (1.10–3.40)	.021
HZO	1.21 (0.87–1.69)	.266	1.91 (0.73–4.97)	.186	0.39 (0.05–2.91)	.355

CNS: central nervous system, HZO: herpes zoster ophthalmicus, OR: odds ratio, CI: confidence interval.

### Association of HZ categories and dementia subtypes

Distinct associations were observed when analyzing specific dementia subtypes (Tables 2 and 3). For AD, the crude OR for any HZ exposure was 1.32 (95% CI: 1.06–1.65). After adjustment for age, sex, and dementia risk factors, the adjusted OR increased to 1.44 (95% CI: 1.14–1.79). This negative confounding suggests the strong positive association between age and AD partially suppressed the unadjusted effect estimate. which did not differ significantly from the association observed for all-cause dementia (*p* = .816). In the type-stratified model, only simple HZ was significantly associated with an increased risk of AD (aOR = 1.49, 95% CI: 1.10–1.98). Associations for other HZ types failed to reach statistical significance due to larger SE. A stronger association was found for VD outcome (compared with all-cause dementia, *p* = .046). The crude OR for any HZ exposure was 1.72 (95% CI: 1.41–2.09), which was further increased after adjusting for the same covariances (aOR = 1.82, 95% CI: 1.49–2.21). No significant interaction between HZ exposure and age groups was observed for either AD or VD (*p* for interaction > .05; Supplementary Table 4). All HZ subtypes except HZO showed significant associations with VD. The aORs were 2.11 (95% CI: 1.17–3.63) for complicated HZ, 1.89 (95% CI: 1.43–2.46) for simple HZ, and 1.76 (95% CI: 1.26–2.43) for CNS-involved HZ.

**Table 3.** Risk of all-cause dementia, Alzheimer’s disease and vascular dementia for herpes zoster with four categories by multivariance logistic analysis.

Variable		All Dementia (N = 390720)		Alzheimer’s Disease (N = 19280)		Vascular Dementia (N = 24621)	
		n	OR (95% CI)	n	OR (95% CI)	n	OR (95% CI)
Exposure							
	No HZ record	380619	ref	18749	ref	24023	ref
	all HZ	10101	1.48 (1.41–1.56)	531	1.44 (1.14–1.79)	598	1.82 (1.49–2.21)
	*Analysis by HZ categories*						
	Simple HZ	5405	1.48 (1.38–1.59)	296	1.49 (1.10–1.98)	305	1.89 (1.43–2.46)
	HZ with CNS-involvement	3258	1.59 (1.46–1.74)	161	1.42 (0.94–2.09)	208	1.76 (1.26–2.43)
	Complicated HZ	1177	1.22 (1.04–1.42)	51	0.95 (0.39–1.99)	67	2.11 (1.17–3.63)
	HZO	261	1.25 (0.88–1.72)	23	2.09 (0.74–5.19)	18	0.41 (0.02–2.03)
Sex							
	Female	192431	ref	11303	ref	11001	ref
	Male	198289	1.11 (1.09–1.13)	7977	1.12 (1.03–1.21)	13620	1.19 (1.10–1.28)
Age at index date							
	50–64	118041	ref	4666	ref	6488	ref
	65–79	221522	1.59 (1.55–1.63)	10255	1.78 (1.59–2.00)	12884	1.72 (1.56–1.90)
	80–102	51157	3.17 (3.08–3.26)	4359	3.31 (2.93–3.75)	5249	3.32 (2.98–3.70)
Number of risk factors*							
	None	254475	ref	12798	ref	16608	ref
	1–2	125823	0.99 (0.97–1.01)	5972	0.59 (0.54–0.65)	7361	0.71 (0.65–0.77)
	>2	10422	1.52 (1.44–1.60)	510	1.14 (0.90–1.42)	652	1.76 (1.46–2.11)

HZ: herpes zoster, CNS: central nervous system, HZO: herpes zoster ophthalmicus, OR: odds ratio, CI: confidence interval.

*Number of risk factor including the diagnosis of hearing impairment, depression, hypertension, alcohol abuse, obesity, diabetes, dyslipidemia, visual impairment, and ischemic heart disease before the index date.

### Sensitivity analysis

The sensitivity analysis included 51,164 dementia patients and 335,461 matched controls, showing identical directions and stronger magnitudes of association for all-cause dementia, AD, and VD (Table 4). For the subtypes of HZ in relation to all-cause dementia, all types except HZO demonstrated significantly increased risks. In the analysis stratified by index date, 56,567 patients with an outcome occurring between 2016 and 2019 showed an aOR of 1.24 (95% CI: 1.02–1.48) for all-cause dementia, whereas 334,153 patients with an index date in 2020 or later had an aOR of 1.51 (95% CI: 1.43–1.59). All sensitivity analyses results remained consistent with the main analysis, reinforcing the positive association between HZ exposure and dementia risk.

**Table 4.** Risk of all-cause dementia, Alzheimer’s disease and vascular dementia for herpes zoster with four categories by multivariance logistics analysis in sensitivity analysis.

Variable		All Dementia (N = 386625)		Alzheimer’s Disease (N = 18671)		Vascular Dementia (N = 23977)		During 2016–2019 (N = 56567)		During 2020–2024 (N = 334153)	
		n	OR (95% CI)	n	OR (95% CI)	n	OR (95% CI)	n	OR (95% CI)	n	OR (95% CI)
Exposure											
	No HZ record	377026	ref	18169	ref	23424	ref	55766	ref	324853	ref
	all HZ	9599	1.59 (1.51–1.67)	502	1.56 (1.24–1.94)	553	2.03 (1.66–2.47)	801	1.24 (1.02–1.48)	9300	1.51 (1.43–1.59)
	*Analysis by HZ categories*										
	Simple HZ	5156	1.58 (1.48–1.70)	287	1.57 (1.16–2.09)	281	2.10 (1.59–2.74)	582	1.22 (0.97–1.51)	4823	1.51 (1.41–1.63)
	HZ with CNS-involvement	3096	1.72 (1.57–1.87)	150	1.57 (1.03–2.31)	197	1.93 (1.38–2.68)	154	1.36 (0.90–2.01)	3104	1.62 (1.48–1.77)
	Complicated HZ	1109	1.32 (1.13–1.55)	48	1.03 (0.42–2.19)	61	2.43 (1.34–4.24)	53	0.90 (0.37–1.89)	1124	1.24 (1.06–1.46)
	HZO	238	1.40 (0.99–1.94)	17	3.26 (1.09–8.90)	14	0.51 (0.03–2.61)	12	2.43 (0.53–8.38)	249	1.22 (0.85–1.70)
Sex											
	Female	190266	ref	10965	ref	10738	ref	28738	ref	163693	ref
	Male	196359	1.11 (1.09–1.13)	7706	1.12 (1.03–1.22)	13239	1.19 (1.11–1.28)	27829	1.31 (1.25–1.38)	170460	1.08 (1.06–1.10)
Age at index date											
	50–64	117196	ref	4574	ref	6387	ref	21452	ref	96589	ref
	65–79	218997	1.59 (1.55–1.63)	9869	1.79 (1.59–2.01)	12499	1.73 (1.56–1.91)	27625	1.59 (1.50–1.69)	193897	1.59 (1.55–1.63)
	80–102	50432	3.18 (3.09–3.27)	4228	3.37 (2.97–3.82)	5091	3.35 (3.01–3.74)	7490	3.14 (2.92–3.38)	43667	3.16 (3.06–3.27)
Number of risk factors											
	None	252954	ref	12469	ref	16275	ref	44216	ref	210259	ref
	1–2	123664	0.99 (0.97–1.01)	5735	0.56 (0.51–0.62)	7096	0.68 (0.63–0.74)	11807	1.51 (1.42–1.60)	114016	0.94 (0.92–0.96)
	>2	10007	1.46 (1.39–1.54)	467	1.02 (0.80–1.30)	606	1.65 (1.35–1.99)	544	1.92 (1.56–2.35)	9878	1.48 (1.40–1.56)

HZ: herpes zoster, CNS: central nervous system, HZO: herpes zoster ophthalmicus, OR: odds ratio, CI: confidence interval.

Two sensitivity analyses were performed. Risks of all-cause dementia, Alzheimer’s disease and vascular dementia for herpes zoster with four categories were calculated by multivariance logistics analysis in first sensitivity analysis which excluded individuals with intellectual disability, or a history of HSV infection or Parkinson’s disease prior to the index date. Second analyses were conducted based on the index date before (2016–2019) and after January 1, 2020 (2020–2025). Risks of all-cause dementia for herpes zoster with four categories were calculated by multivariance logistics analysis.

*Number of risk factor including the diagnosis of hearing impairment, depression, hypertension, alcohol abuse, obesity, diabetes, dyslipidemia, visual impairment, and ischemic heart disease before the index date.

### The temporal impact on the association

**Figure 1.**
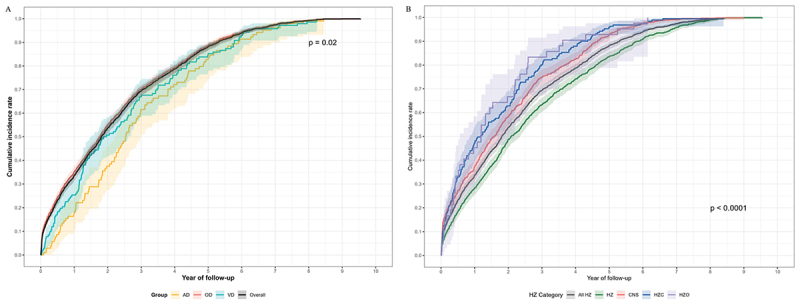
Temporal interval from herpes zoster to dementia diagnosis among patients with both conditions (N = 1,956). This analysis is restricted to dementia cases with a documented history of herpes zoster prior to first dementia diagnosis. The curves depict the cumulative distribution of time from the initial herpes zoster diagnosis to the subsequent dementia diagnosis, stratified by (A) dementia subtype and (B) herpes zoster clinical category. AD: Alzheimer’s disease, OD: unspecific or other dementia, VD: vascular dementia, HZ: simple herpes zoster, CNS: central nervous system, HZC: herpes zoster, HZO: herpes zoster ophthalmicus.

Figure 1 demonstrated the time to dementia diagnosis among 1956 dementia cases who had a documented history of HZ, including 104 AD and 142 VD. This result shows the temporal characteristics from zoster to dementia. The median time from first HZ diagnosis to the confirmation of any type of dementia was 2 y. When stratified by age at HZ onset, the median interval from HZ to dementia diagnosis did not differ significantly across age groups (Kruskal-Wallis test, *p* = .076). By five years after the HZ episode, 88.2% of zoster patients had diagnosed among those eventually became dementia. The median time from HZ onset to dementia diagnosis was shortest for VD (1.5 y), followed by other or unspecific dementia (2.3 y) and AD (2.5 y). The progression interval to dementia varies substantially by HZ subtype (log-rank test, *p* < .001). Patients initially diagnosed with HZO and complicated HZ experienced the most rapid progression. By two years after HZ onset, 66.7% of HZO cases and 62.8% of complicated HZ cases had received a dementia diagnosis. After five years of follow-up, among those with both prior HZ and dementia, 95.8% of patients with complicated HZ and 92.9% with HZO, and 92.9% with CNS-involved had received a dementia diagnosis, compared to 83.6% in the simple HZ group. Pairwise comparisons indicated that the survival curve for simple HZ was significantly different from those of all other HZ types (all *p* < .001), whereas the curves for CNS-involved, complicated, and ophthalmic HZ were not statistically different from each other.

## Discussion

This large-scale, population-based study is the first investigation in China to examine the association between HZ and dementia using EMR, with clinical diagnoses serving as the basis for case definition. Our results revealed that 3.77% of dementia patients had a prior HZ diagnosis within an eight-year medical record, compared to 2.40% of patients without dementia. This indicates that a history of HZ is significantly associated with an increased risk of dementia among individuals aged 50 y and older, with pronounced risk in senior groups. Notably, the risk increased by 82% when specifically considering VD patients.

Our findings align with and extend recent observational studies addressing this association.^10^ Two independent Korean national cohort studies conducted over different 11-year periods consistently demonstrated that untreated VZV infection was associated with an increased risk of dementia (adjusted HR = 1.12, 95% CI: 1.05–1.19), while any VZV infection, regardless of category, increased the risk of all dementia types (HR = 1.41, 95% CI: 1.37–1.46).^22,23^ An Italian cohort including 132,968 individuals over 20 y found that severe zoster increased the risk of dementia compared to a hospitalized population (HR = 1.08, 95% CI: 1.03–1.14), with risk further elevated when compared to the general population (HR = 1.13, 95% CI: 1.07–1.19).^24^ Comprehensive evidence from the United States, analyzing VZV reactivation, recurrent HZ, and herpes zoster vaccination, demonstrated that HZ exposure increases dementia risk and that preventing HZ can reduce dementia incidence. This relationship exhibits a dose-response pattern and temporal characteristics, where dementia incidence increases with the frequency of HZ episodes or with the time elapsed since vaccination.^25^ Our study, based on clinical diagnoses, aligns with these previous findings, with effect sizes consistently falling in the range of 1–2 fold risk range. Collectively, these studies encompassing prevention, diagnosis, control, and treatment of HZ suggest that the epidemiological causal association observed at the population level is unlikely to be coincidental.

Additionally, our study conducted more detailed analyses of dementia and HZ subtypes without altering the overall conclusions. When categorized by VZV reaction sites, patients with CNS involvement had the highest risk of developing dementia, with an increase of nearly 60%, and developed dementia earlier than other categories. This pattern has been similarly observed in several prior studies. A Danish population cohort, while not finding a positive association between all HZ infections and dementia, still identified a two-fold increased dementia risk in cases with CNS involvement.^26^ For VZV infected eyes, compared to no HZ diagnosis, the risk of dementia was increased compared to no HZ diagnosis, but it was not stronger than other infection sites, and the association did not reach statistical significance. This inconsistency was probably attributed to potential differences in disease characteristics across populations, and possibly to the smaller sample size of HZO cases compared to other HZ categories in our study population. Separate analyses were conducted for AD and VD, two common dementia subtypes. An HZ episode was associated with a 1.44-fold risk of AD occurrence, similar to the overall dementia risk, but significantly higher odds of VD by 1.82-fold, within a shorter time span. This pattern contrasts with findings from a Korean study, which reported a higher risk for AD than VD, possibly because that study used VZV infection as the exposure metric, which may differ from clinical diagnosis.^23^

Most existing studies employ a cohort design comparing dementia incidence between groups^6,11,25^; our nested case-control study provides a comparable credibility. The use of EMR data mitigates the major limitation of recall bias inherent in case-control studies and ensures a clear temporal sequence from HZ to dementia outcome. Dementia incidence is often underestimated, as mild symptoms-especially in seniors-may be overlooked, and healthcare-seeking behaviors can distort findings in studies focusing on dementia risk.^27^ Considering the characteristics of dementia, we designed this case-control study from a patient cohort. To reduce detection bias or surveillance bias resulting from differential healthcare utilization between patients and the community population, trauma patients were selected as the control group from underlying patient cohort, whose prevalence theoretically does not differ from the healthy population.^28^ Propensity score matching helps to control strong confounders influencing both HZ and dementia, and by matching on diagnosis date, we accounted for the effects of evolving medical diagnostic standards, coding practices, regional health policies, and population healthcare-seeking behaviors over time. This ensured comparability of baseline characteristics between groups. A pattern suggestive of negative confounding presented in multivariance analysis, indicated the advanced age and established dementia risk factors, are strongly associated with both HZ and dementia. Meanwhile, age-stratified analyses revealed that the association between HZ exposure and dementia was consistently observed across all age groups.

In analyses stratified by index date, the association was evident in both periods. The more pronounced effect observed during the latter period may reflect a combination of factors. On the one hand, the co-circulation of respiratory viruses during the COVID-19 pandemic, including SARS-CoV-2 and influenza, may have contributed to synergistic neuroinflammatory effects when co-occurring with VZV reactivation. On the other hand, disruptions in healthcare-seeking behavior. Some mild patients may have deferred diagnostic evaluation due to restricted healthcare access, while others may have presented earlier owing to seek medical attention earlier.^29^ Although our data cannot definitively differentiate these contributions, the consistency of direction across these distinct temporal contexts supports the results in primary analyses. Another sensitivity analysis excluding patients with a history of HSV and/or Parkinson’s disease enhanced the robustness of our results. Nevertheless, as with all observational studies, residual confounding and the inherent limitations of electronic health record data preclude definitive causal inference. The associations reported herein should be interpreted as evidence of a temporal relationship that warrants further investigation through prospective cohort designs or quasi-experimental studies leveraging vaccine uptake as a natural experiment.

Several hypotheses propose to explain the pathophysiological mechanisms of this association and the risk differences across subtypes. The stronger effect with VD suggests a predominant role for cerebrovascular pathways. VZV can cause vasculopathy, infecting cerebral arteries and leading to inflammation, vascular remodeling, and an increased risk of stroke.^30^ This can directly contribute to vascular cognitive impairment.^31^ Severe HZ, categorized as CNS involvement and complicated HZ, likely represent a greater burden of neuroinflammation and direct vascular damage, explaining the highest risks observed within the VD cohort. The protective effect of the shingles vaccine supports the biological plausibility of this explanation.^32,33^ The shingles vaccine had a lower risk of stroke, and reduced the risk of developing VD by about half.^34,35^ The weaker association with AD might be explained by a more indirect pathway.^25^ The amyloid hypothesis remains a leading theory for AD etiology. On one hand, viral infection inducing systemic inflammation elicit a exacerbates the accumulation of Abeta and tau protein. On the other hand, Experimental studies using cell cultures and animal models collectively support a causative role for HSV-1 in AD neurodegeneration through abnormal beta-amyloid deposition and tau protein accumulation. Experiments have found that cells infected with VZV can activate latent HSV-1 in neurons, leading to AD-like changes. The accumulation of this process gradually slows neuronal signaling, manifesting as cognitive decline progressing to Alzheimer’s disease.

The compelling evidence linking HZ to increased dementia risk, particularly the accelerated cognitive decline following severe HZ episodes, elevates HZ prevention from a clinical priority to a public health imperative with potential neurocognitive benefits. Emerging real-world studies have reported a protective association between HZ vaccination and reduced dementia incidence.^13,32,33,36–39^ Our findings, especially the heightened risk in advanced age groups, provide robust evidence supporting the value of promoting non-NIP vaccine among high-risk populations. By preventing HZ or attenuating its severity, vaccination may serve as a novel, cost-effective strategy to mitigate or delay cognitive decline in the aging population. From a public health perspective, these results underscore the urgency of improving vaccine coverage. In clinical practice, our results suggest that an HZ diagnosis, particularly in older adults, should prompt enhanced cognitive surveillance. Implementing routine cognitive assessments for patients following an HZ episode could facilitate earlier detection of cognitive decline and enable timely intervention, aligning with broader public health promoting of healthy aging.

The context of extremely low HZ vaccination coverage in China provides an important rationale for conducting this study.^40,41^ However, several limitations inherent to EHR-derived data remain unavoidable. Defining study subjects based on clinical disease codes is subject to variations in physician diagnostic accuracy. Although we implemented index date matching, misclassification due to differing medical standards remains possible, particularly for subtyping, where irregular diagnostic categorization created substantial sample size disparities between subgroups. Additionally, subtypes of HZ and dementia relied solely on the primary diagnosis at the first recorded visit, potentially missing new clinical symptoms emerging during multiple subsequent visits throughout the disease course. Furthermore, the case-control design presents challenges for comprehensively analyzing the time-varying effects of HZ exposure on dementia risk. Future research should employ hybrid cohort designs to enable longer-term follow-up observations across multiple stages-from HZ diagnosis or recurrence, as well as natural experiment approaches that leverage the phased rollout of zoster vaccination programs to evaluate whether vaccination modifies dementia risk. In addition, the collection of more granular data on antiviral treatment and medication use would enable evaluation of their potential modifying effects on the association between HZ and dementia.

In summary, this population-based study demonstrates that HZ episodes are associated with the risk of developing all-cause dementia in patients above 50 y old over up to eight years of observation. The particularly strong association with vascular dementia highlights the potential role of cerebrovascular pathways linking viral reactivation to cognitive decline. These findings, viewed in the context of accumulating evidence that HZ vaccination reduces dementia risk, underscore the value of integrating HZ prevention through vaccination into dementia risk-reduction strategies for aging populations, particularly in settings with low coverage.

## Supplementary Material

Supplementary files_revision_clean.docx

## Data sharing

The authors are not permitted to share the individual-level data from the Yichang Healthcare Big Data Platform. The analytic methods are available from the corresponding author upon reasonable request.
