# Supplementary material for: Herpes zoster as a vaccine-preventable risk factor increases the risk of dementia: A nested case-control study in Chinese population
Source: Hum Vaccin Immunother. 2026 Jun 29;22(1):2681253. doi: 10.1080/21645515.2026.2681253 (PMC13327350; doi:10.1080/21645515.2026.2681253)
Supplement: Supplementary files_revision_clean.docx [file KHVI_A_2681253_SM1745.docx]

**supplementary Table 1. Medical conditions with ICD-10 codes and disease categories**

| **Disease Category** | **Condition** | **ICD-10 code*** |
| --- | --- | --- |
| Zoster [herpes zoster] | Zoster encephalitis | B02.0 |
|  | Zoster meningitis | B02.1 |
|  | Zoster with other nervous system involvement | B02.2 |
|  | Zoster ocular disease | B02.3 |
|  | Disseminated zoster | B02.7 |
|  | Zoster with other complications | B02.8 |
|  | Zoster without complication | B02.9 |
| Dementia | Dementia in Alzheimer's disease | F00, G30 |
|  | Vascular Dementia | F01 |
|  | Dementia in other diseases classified elsewhere | F02 |
|  | Unspecified dementia | F03 |
|  | Other degenerative diseases of nervous system, not elsewhere classified | G31, exclude G31.2 |
| Trauma | Injuries to the head | S00-S09 |
|  | Injuries to the neck | S10-S19 |
|  | Injuries to the thorax | S20-S29 |
|  | Injuries to the abdomen, lower back, lumbar spine and pelvis | S30-S39 |
|  | Injuries to the shoulder and upper arm | S40-S49 |
|  | Injuries to the elbow and forearm | S50-S59 |
|  | Injuries to the wrist and hand | S60-S69 |
|  | Injuries to the hip and thigh | S70-S79 |
|  | Injuries to the knee and lower leg | S80-S89 |
|  | Injuries to the ankle and foot | S90-S99 |
|  | Injuries involving multiple body regions | T00-T07 |
|  | Injuries to unspecified parts of trunk, limb or body region | T08-T14 |
|  | Effects of foreign body entering through natural orifice | T15-T19 |
|  | Burns and corrosions | T20-T32 |
|  | Frostbite | T33-T35 |
|  | Poisoning by drugs, medicaments and biological substances | T36-T50 |
|  | Toxic effects of substances chiefly nonmedicinal as to source | T51-T65 |
|  | Other and unspecified effects of external causes | T66-T78 |
|  | Certain early complications of trauma | T79 |
|  | Complications of surgical and medical care, not elsewhere classified | T80-T88 |
|  | Sequelae of injuries, poisoning and of other consequences of external causes | T90-T98 |
| Dementia risk factor | Blind | H30-H35, H53-H54 |
|  | Deaf | H90-H91 |
|  | Dyslipidemia | E78.0-E78.5 |
|  | Depression | F32-F33, F34.1 |
|  | Diabetes mellitus | E10-E14 |
|  | Smoking history | F17, Z91.800x002, Z50.3, Z71.6, T65.2, Z58.7 |
|  | Hypertension | I10-I13, I15 |
|  | Obesity | E65-E66 |
|  | Alcohol consumption | F10, Z50.2, Z71.4, Y90, Y91, T51, K70, G62.1, G31.2, K85.2, K86.0 |
| Underlying medical condition | Cardiovascular disease | I11, I13, I20-I25, I27, I30-I52 |
|  | Cerebrovascular accident | I60-I69,G45 |
|  | Peripheral vascular disease | I70-I89 |
|  | Asthma | J45-J46 |
|  | Chronic obstructive pulmonary disease | J41-J44 |
|  | Chronic kidney disease | E10.2, E11.2,E12.2, E13.2, E14.2, N18-N19, Z49.0, Z49.1, Z99.2, I12, I13 |
|  | Liver disease | K70-K76 |
|  | Systemic lupus erythematosus | M32 |
|  | Rheumatoid arthritis | M05.101, M05.102, M05.3, M06.8, M06.9, M06.000 |
|  | Tumor | C00-C97, D00-D48 |
| All ICD codes were defined according to WHO methods. | | |

**supplementary Table 2. Results of matching variables performed in propensity score matching analyses**

|  | **Before Matching** | | | **After Matching** | | | **Standardized Pair Distance** |
| --- | --- | --- | --- | --- | --- | --- | --- |
|  | **Dementia Patients** | **Trauma Patients** | **SMD** | **Dementia Patients** | **Trauma Patients** | **SMD** |  |
| Number of Patients | 51852 | 820287 |  | 51843 | 338877 |  |  |
| Scaled age, year | 1.0636 | -0.0792 | 1.3403 | 0.8004 | 0.0005 | 0.0143 | 0.0077 |
| Sex, male | 0.4871 | 0.4974 | -0.0206 | 0.4871 | 0.5338 | -0.0935 | 0.0799 |
| Scaled index date | 0.0413 | 0.0018 | 0.0485 | 0.0493 | 0.0471 | 0.0022 | 0.0066 |

PSM: propensity score matching, SMD: standardized mean difference

**supplementary Table 3. Selected baseline characteristics of patients diagnosed with dementia and matched trauma controls**

| **Characteristic** | **Alzheimer's Disease (N=2891)** | **Vascular Dementia (N=3595)** | **Other Dementia (N=45357)** | **p value** |
| --- | --- | --- | --- | --- |
|  | **n** | **n** | **n** |  |
| Sex |  |  |  | 0.001 |
| Male | 1250 (43.24%) | 2051 (57.05%) | 23307 (51.39%) |  |
| Female | 1641 (56.76%) | 1544 (42.95%) | 22050 (48.61%) |  |
| Age at index date (years), mean (std) | 75.2 (9.57) | 74.9 (9.87) | 71.9 (9.25) | <0.001 |
| Age group |  |  |  | <0.001 |
| 50-64 | 414 (14.32%) | 574 (15.97%) | 9415 (20.76%) |  |
| 65-79 | 1454 (50.29%) | 1792 (49.85%) | 26243 (57.86%) |  |
| 80-102 | 1023 (35.39%) | 1229 (34.19%) | 9699 (21.38%) |  |
| Ethnicity |  |  |  | <0.001 |
| Han | 1851 (93.25%) | 2349 (96.51%) | 28481 (86.73%) |  |
| Minority | 134 (6.75%) | 85 (3.49%) | 4359 (13.27%) |  |
| Education background |  |  |  | <0.001 |
| Junior high school and below | 1170 (70.31%) | 1488 (71.61%) | 21815 (76.23%) |  |
| High (Vocational) school education | 371 (22.3%) | 438 (21.08%) | 5009 (17.5%) |  |
| Bachelor's degree or above | 123 (7.39%) | 152 (7.31%) | 1793 (6.27%) |  |
| Marital status |  |  |  | <0.001 |
| Married | 1536 (82.98%) | 1924 (85.02%) | 26060 (83.04%) |  |
| Discoverture (single, widowed or divorced) | 315 (17.02%) | 339 (14.98%) | 5321 (16.96%) |  |
| Disabled |  |  |  | <0.001 |
| Intelligence disability | 1 (0.03%) | 0 (0%) | 24 (0.05%) |  |
| Other disabilities | 13 (0.45%) | 31 (0.86%) | 1155 (2.55%) |  |
| None | 2877 (99.52%) | 3564 (99.14%) | 44179 (97.4%) |  |
| Exposure |  |  |  | 0.003 |
| Simple HZ | 59 (2.04%) | 75 (2.09%) | 915 (2.02%) |  |
| HZ with CNS-involvement | 32 (1.11%) | 49 (1.36%) | 593 (1.31%) |  |
| Complicated HZ | 7 (0.24%) | 17 (0.47%) | 167 (0.37%) |  |
| HZO | 6 (0.21%) | 1 (0.03%) | 35 (0.08%) |  |
| None | 2787 (96.4%) | 3453 (96.05%) | 43647 (96.23%) |  |
| Age at herpes zoster (years), mean (std) | 75.3 (8.60) | 74.0 (9.64) | 71.2 (9.17) | <0.001 |
| Antiviral drug |  |  |  | 0.013 |
| Yes | 46 (1.59%) | 73 (2.03%) | 898 (1.98%) |  |
| No | 2845 (98.41%) | 3522 (97.97%) | 44459 (98.02%) |  |
| Length of follow up (years), mean (std) | 2.93 (2.00) | 2.55 (2.08) | 2.24 (2.00) | 0.001 |
| Underlying medical condition |  |  |  |  |
| Smoking history | 86 (2.97%) | 114 (3.17%) | 2437 (5.37%) | <0.001 |
| Alcohol consumption | 46 (1.59%) | 84 (2.34%) | 1911 (4.21%) | <0.001 |
| Obesity | 0 (0%) | 3 (0.08%) | 21 (0.05%) | 0.045 |
| Hypertension | 558 (19.3%) | 850 (23.64%) | 12906 (28.45%) | <0.001 |
| Diabetes mellitus | 238 (8.23%) | 372 (10.35%) | 4067 (8.97%) | <0.001 |
| Dyslipidemia | 151 (5.22%) | 232 (6.45%) | 2661 (5.87%) | 0.002 |
| Depression | 44 (1.52%) | 45 (1.25%) | 240 (0.53%) | <0.001 |
| Blind | 61 (2.11%) | 70 (1.95%) | 812 (1.79%) | 0.118 |
| Deaf | 35 (1.21%) | 23 (0.64%) | 447 (0.99%) | <0.001 |
| Cardiovascular disease | 204 (7.06%) | 321 (8.93%) | 2872 (6.33%) | <0.001 |
| Cerebrovascular accident | 538 (18.61%) | 871 (24.23%) | 8245 (18.18%) | <0.001 |
| Peripheral vascular disease | 45 (1.56%) | 74 (2.06%) | 586 (1.29%) | <0.001 |
| COPD | 280 (9.69%) | 336 (9.35%) | 5222 (11.51%) | <0.001 |
| Chronic kidney disease | 74 (2.56%) | 109 (3.03%) | 947 (2.09%) | 0.347 |
| Liver disease | 99 (3.42%) | 144 (4.01%) | 2179 (4.8%) | <0.001 |
| Tumor | 82 (2.84%) | 106 (2.95%) | 1678 (3.7%) | <0.001 |
| Herpes simplex virus | 12 (0.42%) | 8 (0.22%) | 133 (0.29%) | 0.002 |
| Parkinson | 57 (1.97%) | 64 (1.78%) | 384 (0.85%) | <0.001 |
| Number of risk factor* |  |  |  | <0.001 |
| 0 | 2123 (73.43%) | 2515 (69.96%) | 28000 (61.73%) |  |
| 1-2 | 669 (23.14%) | 909 (25.29%) | 15565 (34.32%) |  |
| >2 | 99 (3.42%) | 171 (4.76%) | 1792 (3.95%) |  |

HZ: herpes zoster, CNS: central nervous system, HZO: herpes zoster ophthalmicus, COPD: chronic obstructive pulmonary disease

*Number of risk factor including the diagnosis of hearing impairment, depression, hypertension, alcohol abuse, obesity, diabetes, dyslipidemia, visual impairment, and ischemic heart disease before the index date.

**supplementary Table 4. Risk of all-cause dementia, Alzheimer's disease and vascular dementia for herpes zoster with age-stratified by multivariance logistic analysis**

| **Variable** | | **All Dementia (N=390720)** | | **Alzheimer's Disease (N=19280)** | | **Vascular Dementia (N=24621)** | |
| --- | --- | --- | --- | --- | --- | --- | --- |
|  |  | **n** | **OR (95% CI)** | **n** | **OR (95% CI)** | **n** | **OR (95% CI)** |
| Exposure |  |  |  |  |  |  |  |
|  | No HZ record | 380619 | ref | 18749 | ref | 24023 | ref |
|  | all HZ | 10101 | 1.77 (1.56-2.00) | 531 | 0.93 (0.39-1.89) | 598 | 2.13 (1.23-3.48) |
| Exposure* Age at index date | | | |  |  |  |  |
|  | HZ record at 65-79 | 6361 | 0.78 (0.68-0.90) | 300 | 1.37 (0.62-3.44) | 326 | 0.88 (0.50-1.61) |
|  | HZ record at 80-102 | 1664 | 0.87 (0.74-1.03) | 141 | 2.03 (0.91-5.18) | 164 | 0.77 (0.42-1.46) |
| Sex |  |  |  |  |  |  |  |
|  | Female | 192431 | ref | 11303 | ref | 11001 | ref |
|  | Male | 198289 | 1.11 (1.09-1.13) | 7977 | 1.12 (1.03-1.21) | 13620 | 1.19 (1.10-1.28) |
| Age at index date | |  |  |  |  |  |  |
|  | 50-64 | 118041 | ref | 4666 | ref | 6488 | ref |
|  | 65-79 | 221522 | 1.60 (1.56-1.64) | 10255 | 1.77 (1.58-1.99) | 12884 | 1.73 (1.56-1.91) |
|  | 80-102 | 51157 | 3.18 (3.09-3.27) | 4359 | 3.25 (2.87-3.68) | 5249 | 3.35 (3.00-3.74) |
| Number of risk factors* | |  |  |  |  |  |  |
|  | None | 254475 | ref | 12798 | ref | 16608 | ref |
|  | 1-2 | 125823 | 0.99 (0.97-1.01) | 5972 | 0.59 (0.54-0.65) | 7361 | 0.71 (0.65-0.77) |
|  | >2 | 10422 | 1.52 (1.44-1.60) | 510 | 1.14 (0.91-1.43) | 652 | 1.75 (1.46-2.10) |

HZ: herpes zoster, CNS: central nervous system, HZO: herpes zoster ophthalmicus, OR: odds ratio, CI: confidence interval.

*Number of risk factor including the diagnosis of hearing impairment, depression, hypertension, alcohol abuse, obesity, diabetes, dyslipidemia, visual impairment, and ischemic heart disease before the index date.
